# Early rehabilitation for volumetric muscle loss injury augments endogenous regenerative aspects of muscle strength and oxidative capacity

**DOI:** 10.1186/s12891-018-2095-6

**Published:** 2018-05-29

**Authors:** Sarah M. Greising, Gordon L. Warren, W. Michael Southern, Anna S. Nichenko, Anita E. Qualls, Benjamin T. Corona, Jarrod A. Call

**Affiliations:** 1 Extremity Trauma and Regenerative Medicine, United States Army Institute of Surgical Research, Fort Sam Houston, Texas, 78234 USA; 2 Department of Physical Therapy, Byrdine F. Lewis School of Nursing and Health Professions, Georgia State University, Atlanta, GA 30302 USA; 3 Department of Kinesiology, University of Georgia, Athens, GA 30602 USA; 4 Regenerative Bioscience Center, University of Georgia, Athens, GA 30602 USA

**Keywords:** Electrical stimulation, Neuromusculoskeletal injury, Regenerative medicine, Orthopaedic trauma, Skeletal muscle injury, Range of motion

## Abstract

**Background:**

Volumetric muscle loss (VML) injuries occur due to orthopaedic trauma or the surgical removal of skeletal muscle and result in debilitating long-term functional deficits. Current treatment strategies do not promote significant restoration of function; additionally appropriate evidenced-based practice physical therapy paradigms have yet to be established. The objective of this study was to develop and evaluate early rehabilitation paradigms of passive range of motion and electrical stimulation in isolation or combination to understand the genetic and functional response in the tissue remaining after a multi-muscle VML injury.

**Methods:**

Adult male mice underwent an ~ 20% multi-muscle VML injury to the posterior compartment (gastrocnemius, soleus, and plantaris muscle) unilaterally and were randomized to rehabilitation paradigm twice per week beginning 2 days post-injury or no treatment.

**Results:**

The most salient findings of this work are: 1) that the remaining muscle tissue after VML injury was adaptable in terms of improved muscle strength and mitigation of stiffness; but 2) not adaptable to improvements in metabolic capacity. Furthermore, biochemical (i.e., collagen content) and gene (i.e., gene arrays) assays suggest that functional adaptations may reflect changes in the biomechanical properties of the remaining tissue due to the cellular deposition of non-contractile tissue in the void left by the VML injury and/or differentiation of gene expression with early rehabilitation.

**Conclusions:**

Collectively this work provides evidence of genetic and functional plasticity in the remaining skeletal muscle with early rehabilitation approaches, which may facilitate future evidenced-based practice of early rehabilitation at the clinical level.

**Electronic supplementary material:**

The online version of this article (10.1186/s12891-018-2095-6) contains supplementary material, which is available to authorized users.

## Background

Volumetric muscle loss (VML) is a debilitating orthopaedic condition that results in chronic functional deficits and disability [1–3]. With no current surgical or rehabilitative standard of care to address the soft tissue loss, VML injures are left to follow the natural sequela of injury that ultimately results in the replacement of contractile skeletal muscle with non-contractile pathologic fibrotic tissue [4]. Furthermore, functional capacity after VML injury continues to deteriorate over time [5]. As such, interventions and treatment approaches are urgently needed to ameliorate the progressive functional disability related to VML injury [6].

Physical therapy and rehabilitation are an important component of functional improvements following all neuromusculoskeletal injuries, but there is currently a dearth of experimental studies to support any evidenced-based practice for VML injury. In part, the lack of clinical rehabilitation guidelines could be related to heterogeneous injury pattern and the prevalence of the multiple concomitant injuries to VML, such as fracture [7]. A limited number of VML injury case studies have consistently observed chronic functional deficits and disability that were not ameliorated by delayed, prolonged, intensive physical therapy [2, 8–10]. Lack of human-based experimental studies have been addressed using small and large animal models [6]. In fact, a small number of pre-clinical studies involving rodent models have demonstrated beneficial functional remodeling of skeletal muscle with running as a physical therapy modality [11–14]. However, quadruped running may rely upon and engage muscles differently than biped running, so more refined experimental designs are necessary to validate actively engaging VML injured muscle as part of a rehabilitation approach.

Understanding of early rehabilitative interventions in VML patients has yet to be fully established. Early mobilization and rehabilitation initiated in the hospital setting for various other clinical conditions results in shorter admission times and improved function [15]. Passive range of motion exercise are non-weight bearing rehabilitation techniques that do not rely on functionally innervated muscle fibers. The tolerance and effectiveness of range of motion rehabilitation to assist in recovery from conditions ranging from contraction-induced muscle injury [16] to rotator cuff repair [17] has been documented. It is expected that passive movement may mitigate the muscle stiffness following VML injury, but the approach has not been tested. Interestingly, in Duchenne muscular dystrophy, a condition presenting with pathologic fibrosis, muscle weakness and stiffness, muscle activation regimens in combination with range of motion therapy are reportedly more effective in improving limb endurance and function compared to range of motion therapy alone clinically [18]. VML injuries may present with motor unit recruitment issues, either from loss of consciousness (e.g., prolonged comatose state), peripheral nerve damage, or damaged neural tracks, and therefore a potential rehabilitation technique to circumvent these limitations is intermittent electrical stimulation. Intermittent electrical stimulation will recruit the muscle fibers remaining following injury via subdermal stimulation. Electrical stimulation has been shown to promote motor and sensory reinnervation and regeneration following nerve injury [19], and induce hyperopic overload [20, 21]. Both range of motion and intermittent electrical stimulation techniques represent rehabilitation paradigms that conceivably could be conducted in a hospital setting, even while patients were non-weight bearing.

This work sought to develop and evaluate two early rehabilitation paradigms of passive range of motion and electrical stimulation to understand the genetic and functional response in the tissue remaining following a multi-muscle VML injury. We developed the rehabilitation protocols broadly off of previous work [16, 18, 20, 21]. We hypothesized that early intervention following VML injury would enhance the endogenous regenerative and oxidative capacity of the muscle remaining following VML injury, ultimately improving muscle function.

## Methods

### Animals

Adult male C5BL/6 mice (*n* = 96) were purchased from Jackson Laboratories (Bar Harbor, ME). Animals were maintained on a 12 h light-dark schedule under specific pathogen-free conditions with ad libitum food and water in a vivarium accredited by the American Association for the Accreditation of Laboratory Animal Care. Upon arrival, all mice were given at least 1 week to acclimate to the facility prior to any experimentation. All protocols and animal care guidelines were approved by the Institutional Animal Care and Use Committee at the United States Army Institute of Surgical Research (A16–036) or the University of Georgia (A2017 08–004), in compliance with National Institute of Health Guidelines. All components were conducted in compliance with the Animal Welfare Act, the Implementing Animal Welfare Regulations and in accordance with the principles of the Guide for the Care and Use of Laboratory Animals.

At ~ 12.5 weeks of age mice underwent a VML injury to the posterior compartment of the hindlimb and were randomized to various treatment groups. Specific groups received no-treatment (VML), rehabilitation interventions of range of motion exercise (ROM) or range of motion and intermittent electrical stimulation (ROM-E). All rehabilitation bouts were conducted two times per week, beginning 2 days post-injury for up to 14 days post-injury for the acute study or 4 months for the chronic study. A subset of uninjured (naïve, no surgical intervention) control mice was used for various analyses throughout the project. Tissue harvest was conducted ~ 24 h following the final rehabilitation bout while mice were deeply anesthetized with isoflurane (1.5–2.0%) and mice were euthanized with an injection of Fatal Plus (150 mg/kg; intra-cardiac) while still under anesthesia. Following VML surgery all mice recovered promptly and displayed only slight limitations in mobility. No unexpected deaths or adverse outcomes were noted in any group across the 4 months evaluated.

### Volumetric muscle loss (VML) surgery

While anesthetized (isoflurane 1.5–2.0%) a surgical VML was created on the middle third of the posterior compartment using a surgical approach modified from previous work [22]. All mice received a pre-surgical (~ 30 min prior) administration of buprenorphine-SR (1.2 mg/kg; s.c.) for pain management. Briefly, a posterior-lateral incision was made through the skin to reveal the gastrocnemius muscle. Blunt and specific dissection of the skin, fascia, and hamstring muscle was used to reveal the posterior aspect of the gastrocnemius muscle. Blunt dissection was used to isolate the muscle compartment off the dorsal aspect of the tibia and a small metal plate was inserted below the deep soleus muscle but above the tibia and a punch biopsy (4 mm, approximately 20% volume loss of muscle; *see* Table 1) was performed through the middle third of the muscle compartment. Any bleeding was stopped with light pressure. Following the surgical injury the skin incision was closed with simple interrupted suture (6–0 Silk). In all cases the left limb underwent the VML injury and the contralateral was used as an injured intra-animal control for biochemical and gene expression analysis.

**Table 1:** Multi-muscle volumetric muscle loss injury

	*n*	VML defect mass (mg)	Injured: uninjured gastrocnemius muscle mass	Force deficit from control (%)
3 days	4	18.6 ± 1.3	0.94 ± 0.09	–
7 days	4	18.8 ± 1.0	0.88 ± 0.08	–
14 days	4	19.1 ± 1.3	0.66 ± 0.02*	–
1 month	6	20.2 ± 0.5	0.77 ± 0.05	- 62.5 ± 3.2†
2 months	6	18.2 ± 0.9	0.88 ± 0.06	- 61.8 ± 4.7
4 months	6	19.8 ± 0.8	0.90 ± 0.02	- 51.0 ± 2.7
*P*-value		0.592	0.029	0.043

Mean ± SE; Significantly different than *3 days or †4 months post-VML

### Rehabilitation

**Fig. 1:**
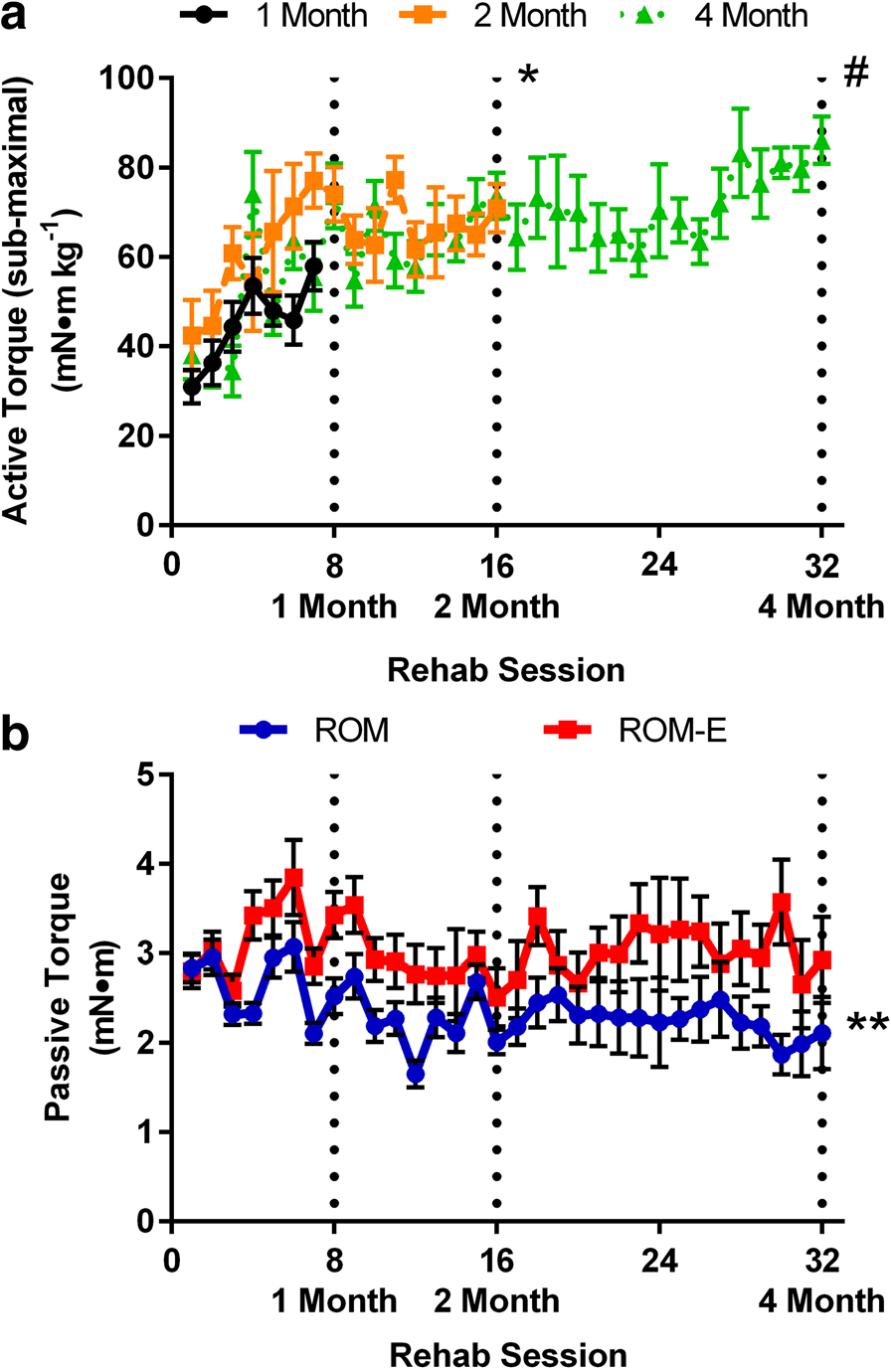
Effect of rehabilitation session number on active and passive torque about the ankle joint. **a** Sub-maximal active torques (20 Hz) were used to identify optimal stimulation parameters during each rehabilitation session, and there was a positive relationship between the number of rehabilitation sessions and active torque of the ankle plantar flexor muscles. *Torque was greater at session #16 (2 Month) compared to session #8 (1 Month) (*P* < 0.05). # Torque was greater at session #32 (4 Month) compared to session #8 (1 Month) (*P* < 0.05). **b** Passive torque about the ankle joint was assessed at 20° dorsiflexion, and passive torque decreased over time (session 1 compared to session 32) for ROM mice, but there was no change in passive torque over time in ROM-E mice. **ROM session 32 > ROM session 1 (*P* < 0.05)

All rehabilitation sessions were conducted while the mouse was anesthetized (isoflurane 1.5–2.0%) and body temperature was maintained. At each bout the knee and foot of the left limb was stabilized at 90^o^ (neutral position) and the foot was secured to a foot plate attached to a servomotor (300B-LR, Aurora Scientific, Aurora, Ontario, Canada). Under computer control, the servomotor passively rotated the ankle 40° through dorsi- and plantar-flexion, specifically 20° from neutral for both directions. Continuous range of motion was conducted for 30 min with each set taking 5 s, followed by a 5 s rest period at neutral for the range of motion alone groups. For groups that received combined range of motion and intermittent electrical stimulation, stimulation occurred during rest phases of the range of motion protocol. Stimulation was elicited using platinum-iridium (Pt-Ir) needle electrodes positioned percutaneously on either side of the sciatic nerve (S48 and SIU5, Grass Technologies, West Warwick, RI, USA). Progressive stimulation parameters were utilized to promote continuous adaptation and were as follows: 30 Hz, 50% duty cycle (immediate post-injury to 1 month); 45 Hz, 25% duty cycle (1 month to 2 months); and 80 Hz, 12.5% duty cycle (2 months to 4 months). These parameters were selected based on the following rationale: 1) 30, 45, and 80 Hz represent the linear phase of the torque-frequency relationship and reflect ~ 25, 50, and 75% peak-isometric torques in uninjured muscle [23, 24]; and 2) a reduced duty cycle for higher-frequencies contractions minimized potential for fatigue. All rehabilitation occurred twice per week throughout the study period, specifically for the acute study on days 2, 6, 9, and 13 days post-surgery. During rehabilitation sessions, ideal electrode placement and current (mAmps) were validate by a series of sub-maximal 20 Hz stimulations. These sub-maximal active torque (ROM-E group only), as well as passive torques (both ROM and ROM-E groups) about the ankle joint was evaluated post-hoc as an assessment of ongoing adaptation to the rehabilitation strategy (*see* Fig. 1).

### Muscle function

In vivo maximal isometric torque of the ankle plantarflexors (gastrocnemius, soleus, and plantaris muscle) was assessed as previously described [23–25] and was determined at the terminal time point. Briefly, mice were anesthetized using 2% isoflurane in oxygen, and then the left hindlimb was depilated and aseptically prepared, the foot placed in a foot-plate attached to a servomotor (Model 300C-LR; Aurora Scientific, Aurora, Ontario, Canada), and Pt-Ir needle electrodes (Grass Technologies, West Warwick, RI, USA) were inserted percutaneously on either side of the nerve. To avoid recruitment of the anterior crural muscles responsible for dorsiflexion, the common perineal nerve was severed [26]. Peak isometric torque was achieved by varying the current delivered to the sciatic nerve which branches to the tibial nerve thus innervating the ankle plantarflexor muscles. To account for differences in body size among mice, torques (mN●m) was normalized by body mass (kg).

### Hydroxyproline

Content of hyrdoxyproline in the muscle was used to determine collagen content following injury. Content was determined biochemically as previously described [27, 28].

### Gene expression

At the time of tissue harvest the gastrocnemius were excised and placed in TRIzol and snap frozen in liquid nitrogen and stored at -80 °C until analysis. RNA was isolated and gene expression was analyzed using a custom designed gene array (RT^2^ Profiler PCR Array; Qiagen) with genes related to myogenic, metabolic, fibrotic, inflammatory, and neural (Additional file 1: Table S1) response to injury per manufacture’s instruction. Data was processed with GAPDH as the endogenous control and expression was calculated relative to contralateral control muscle or the non-repaired VML injured muscle at the same time point, as appropriate and noted. Differentially expressed genes were analyzed with iPathway using a fold change of 0.6 and adjusted *P*-value of 0.05 thresholds.

### Mitochondrial

Immediately following dissection, portions of the medial and lateral gastrocnemius muscles from uninjured and injured limbs were dissected on a chilled aluminum block in 4 °C buffer X (7.23 mM K_2_EGTA, 2.77 mM Ca K_2_EGTA, 20 mM imidazole, 20 mM taurine, 5.7 mM ATP, 14.3 mM PCr, 6.56 mM MgCl_2_-6H_2_O, 50 mM k-MES) into thin muscle fiber bundles as reported previously [29]. Permeabilization of muscle fibers was achieved by transferring fibers to a vial containing buffer X and saponin (50 μg/ml) and incubating (i.e., gentle rocking) at 4 °C for 30 min. Muscle fiber bundles were rinsed for 15 min in buffer Z (105 mM k-MES, 30 mM KCl, 10 mM KH_2_PO_4_, 5 mM MgCl_2_, 0.5 mg/ml BSA, 1 mM EGTA) at 4 °C. All measurements were performed using a Clark-type electrode (Oxygraph Plus System, Hansatech Instruments, UK) at 25 °C. Prior to each experiment, the electrode was calibrated according to the manufacturer’s instructions and 1 ml of O_2_ infused buffer Z was added to the chamber. Muscle fiber bundles were weighed (~ 2.5 mg for all samples) and added to the chamber. State 4 respiration (leak respiration in the absence of ADP) was initiated by the addition of glutamate (10 mM) and malate (5 mM). State 3 respiration (respiration coupled to ATP synthesis) was initiated by the addition of ADP (2.5 mM) and succinate (10 mM). Cytochrome c (10 μM) was added to measure the integrity of the outer mitochondrial membrane. State 3 uncoupled respiration (respiration uncoupled from ATP synthesis) was initiated by the addition of FCCP (0.5 μM). Mitochondrial respiration was terminated by the addition of cyanide (250 mM).

### Statistical analysis

All data was analyzed using JMP (version 10.0 SAS Institute, Inc., NC). Data was analyzed separately using a variety of ANOVAs, when appropriate Tukey HSD post-hoc analysis was performed. Data are reported as mean ± SE, unless otherwise specified and significance was accepted at *P* < 0.05.

## Results

### Multi-muscle volumetric muscle loss (VML) injury

VML injury in military [30, 31] and civilian [32] populations commonly involve 2 or more muscles. To date most VML injury models have been to an isolated muscle, with only a limited number to multiple muscles within the quadriceps [33, 34]. Therefore, our first goal was to establish a murine multi-muscle VML injury model. Because the plantarflexor muscles within the posterior compartment of the rodent hind leg are highly recruited during normal ambulation and are weight bearing [35], this muscle group is ideal for rehabilitation studies. A full-thickness VML injury was created through the plantarflexor gastrocnemius, plantaris, and soleus muscles (Table 1) at the tibia mid-diaphyseal level, resulting in the removal of ~ 19 mg of tissue or ~ 20% of the combined plantarflexor muscle wet weight. The partial tissue resection caused an ~ 50% maximal isometric force loss and ~ 2 fold increase in passive torque (muscle stiffness) about the ankle through 4 months post-injury, indicating successful creation of a model that recapitulates pathophysiological aspects of VML injury in patients [2, 9].

### Early rehabilitation

To validate early rehabilitation approaches, 2 days post-VML injury, mice were randomly assigned to one of the following groups: VML alone (VML), passive range of motion (ROM), or ROM plus intermittent electrical stimulation (ROM-E). Range of motion rehabilitation involved passively moving the ankle joint through 40° of motion and the intent was to reduce muscle stiffness associated with the deposition of collagens in and around the VML injury site. Intermittent electrical stimulation rehabilitation involved recruitment of the ankle plantar flexor muscles via sciatic nerve stimulation with Pt-Ir needle electrodes. The intent was to enhance strength by activating the remaining muscle after VML injury, during a time in which significant motoneuron axotomy is present following injury [36]. Rehabilitation strategies (twice weekly for 30 min) were continued in different cohorts of mice for 1, 2, or 4 months post-VML (*n* = 6 mice/group/time). A small cohort of completely uninjured mice was included to observe deficits associated with the VML injury and the relative recovery with early rehabilitation therapy (*n* = 8 mice).

### Functional response to early rehabilitation

To determine if early rehabilitation approaches were beneficial, functional responses were analyzed at each rehabilitation bout. First, in both the ROM and ROM-E groups, passive torque about the ankle joint was recorded and analyzed during each therapy session. Additionally at each session, sub-maximal active torque about the ankle joint was evaluated in the ROM-E group only. There was a positive association between the number of rehabilitation sessions and sub-maximal plantar flexor muscle torque about the ankle (Main Effect Time, *P* < 0.001, Fig. 1), and overall torque was ~ 125% greater at the last compared to the first session. There was a significant interaction between group and time for passive torque about the ankle joint (*P* = 0.034), as passive torque decreased 25% with range of motion rehabilitation but was not changed over time with combined range of motion and electrical stimulation (Fig. 1). Collectively, these inter-rehabilitation session analyses demonstrated on-going functional remodeling of the injured limb.

Injured and contralateral uninjured gastrocnemius muscle masses were recorded to determine the long-term effect of injury and early rehabilitation on muscle atrophy and possible hypertrophy. There was no effect of early rehabilitation on injured gastrocnemius muscle mass relative to uninjured across time; however, independent of group, the relative mass was 18% greater at 4 months compared to 1 month (Main Effect Time, *P* = 0.025, Additional file 2: Figure S1). Body mass was not affected by early rehabilitation (Additional file 2: Figure S1).

**Fig. 2:**
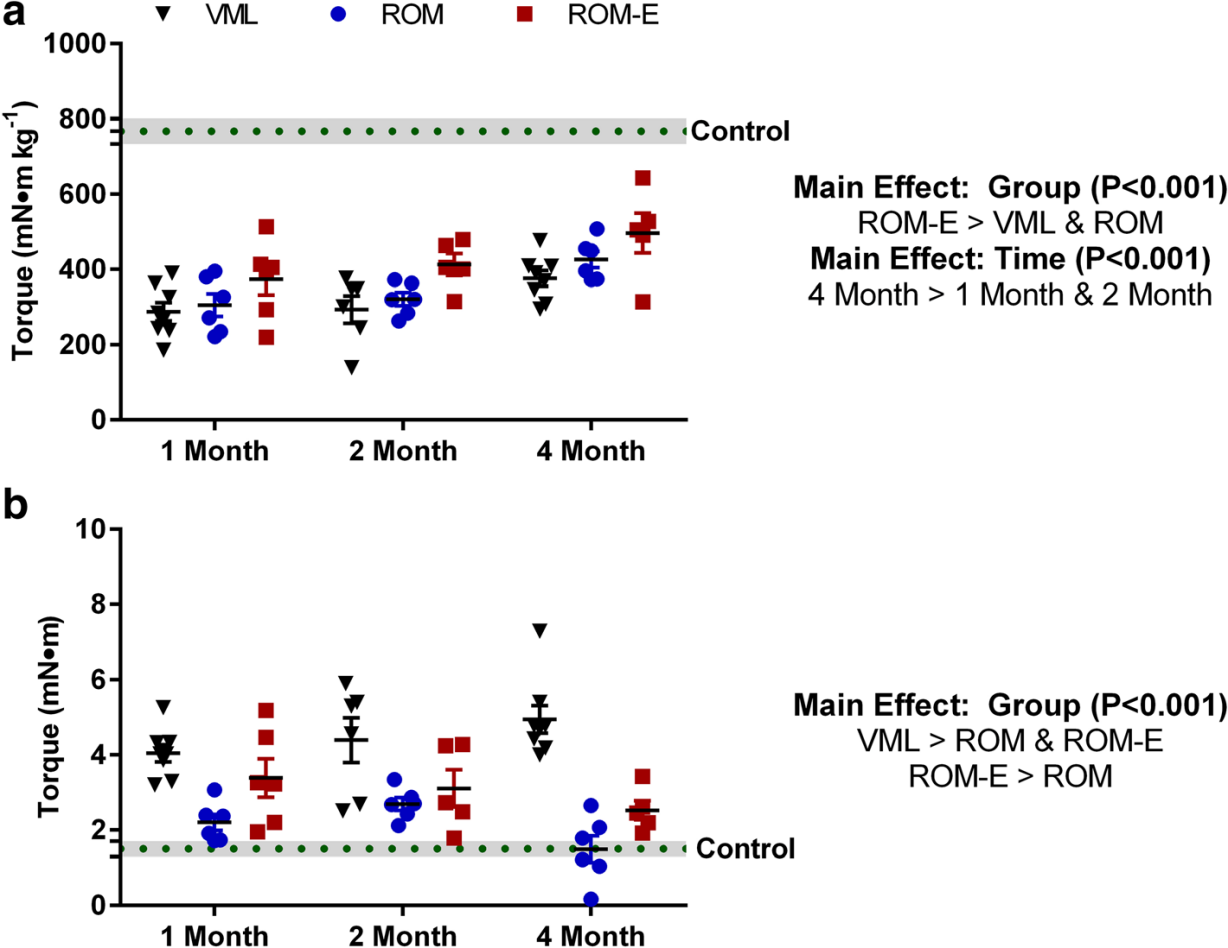
Effect of VML injury and rehabilitation on study endpoint active and passive torque about the ankle joint. **a** Peak isometric torque of the ankle plantar flexor muscles was greater following ROM-E rehabilitation compared to VML-alone and ROM rehabilitation, independent of time. Control = 768 ± 34 mN●m kg ^− 1^. **b** Passive torque of the ankle plantar flexor muscles at 20° dorsiflexion was greatest in VML-along compared to ROM and ROM-E rehabilitation, and lowest following ROM rehabilitation. Control = 1.5 ± 0.2 mN●m

At 1, 2 or 4 months post-VML injury, peak isometric torque of the ankle plantar flexor muscles was assessed to determine contractile function. Independent of time, peak isometric plantar flexor muscle torque was greater in ROM-E mice compared to VML and ROM mice (32 and 21%, respectively; Main Effect Group, *P* < 0.001, Fig. 2 and Additional file 3: Figure S2). At 4 months, VML injury represented a 51% deficit in torque (Control: 768 ± 34 mN●m kg^− 1^ vs. VML: 376 ± 21 mN●m kg^− 1^; *see* Table 1), and while ROM-E mice were stronger than VML mice, a 35% deficit remained (Control: 768 ± 34 mN●m kg^− 1^ vs. ROM-E: 496 ± 118 mN●m kg^− 1^). Collectively, rehabilitation using ROM-E gave rise to functional improvements but was not able to completely mitigate VML-related functional deficits.

Passive torque at 20° dorsiflexion (i.e., when plantar flexor muscles are passively resisting the stretch) was assessed to determine muscle stiffness. There was a strong trend for a significant interaction (*P* = 0.056). At 4 months, VML injury resulted in over a 3-fold increase in passive stiffness (Control: 1.5 ± 0.2 mN●m vs. VML: 4.9 ± 0.4 mN●m), but early ROM rehabilitation attenuated this effect. Independent of time, passive torque of the plantar flexor muscles following ROM and ROM-E rehabilitation were less compared to VML mice (− 52% and − 32%, respectively), and ROM resulted in 29% less passive torque compared to ROM-E (Main Effect Time, *P* < 0.001, Fig. 2).

**Fig. 3:**
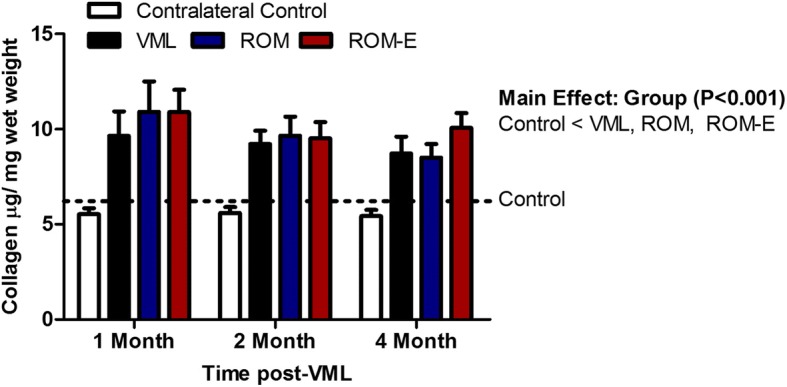
Effect of VML injury and rehabilitation on gastrocnemius muscle collagen content. Gastrocnemius muscle collagen content was greater in uninjured compared to contralateral uninjured control limbs, independent of time and rehabilitation group (*P* < 0.001). Control = 6.21 ± 0.59 μg collagen per mg muscle wet weight

Collagen content of the gastrocnemius muscles was measured since passive stiffness was greater with VML injury. There was a significant effect of injury, independent of time, as total collagen content was ~ 2-fold greater in injured limbs of VML, ROM, & ROM-E mice compared to uninjured limbs (*P* < 0.001, Fig. 3). While there were noted improvements in passive torque about the ankle within the muscle following ROM rehabilitation the collagen deposition and expected fibrotic deposition remained unchanged.

### Oxidative response to early rehabilitation

**Fig. 4:**
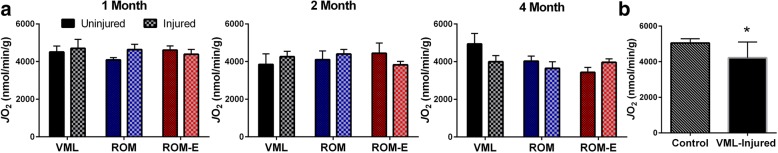
Effect of VML injury and rehabilitation on mitochondrial function of permeabilized muscle fibers. **a** There was no effect of time, group, or VML injury on oxygen consumption rates of permeabilized muscle fibers for VML-injured muscles. **b** Oxygen consumption rates of permeabilized muscle fibers was significantly greater in completely uninjured mice (Controls) compared to VML-injured mice (both injured and contralateral uninjured limbs pooled)

To determine the metabolic function of the remaining muscle tissue after VML injury, oxygen consumption rates for permeabilized fibers isolated adjacent to the injury site were compared to fibers from the contralateral uninjured limb. There was no significant interaction or main effects (*P* ≥ 0.112, Fig. 4). However, independent of time and group, oxygen consumption rates were 25% greater in fibers from completely uninjured mice compared to mice that had a unilateral VML injury (Control: 5054 ± 233 nmol/min/g vs. VML-Injured: 4225 ± 87.39 nmol/min/g, *P* < 0.001, Fig. 4). This signals a previously undescribed impairment with VML injury, which may reflect metabolic maladaptation to changes in muscle recruitment that was resistant to rehabilitative approaches explored in this study.

### Acute genetic response to volumetric muscle loss injury

**Fig. 5:**
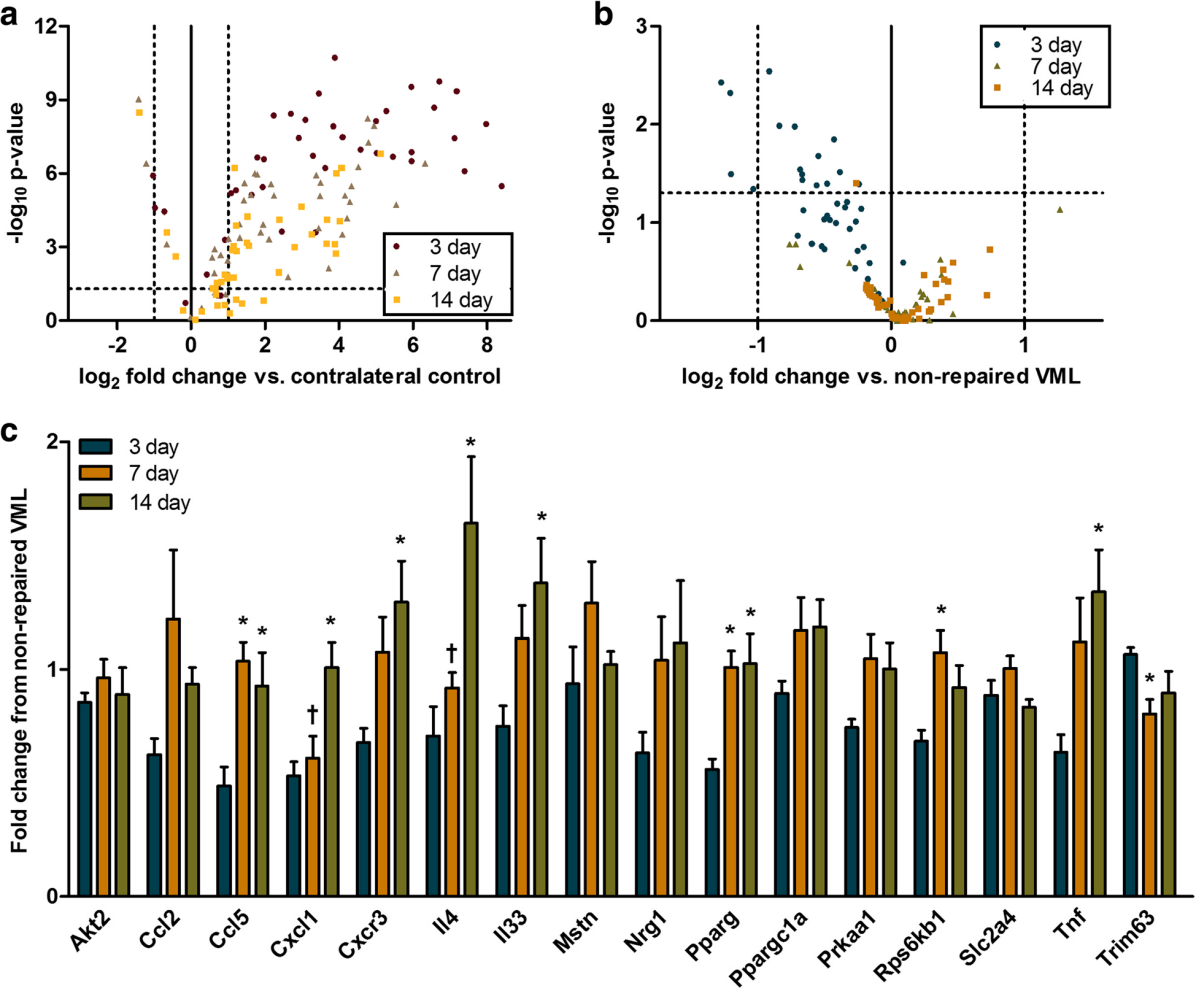
Custom designed (inflammatory, neurogenic, fibrotic, myogenic, and metabolic, see Additional file 1: Table S1) PCR array analysis presents a significant change in regulation following VML injury and early rehabilitation. **a** The response to VML injury was assessed at 3, 7 and 14 days post-VML compared to contralateral control muscle. **b** The response to early rehabilitation was compared to non-repaired VML at the same time point (3, 7, or 14 days post-VML), treatment groups were collapsed. The dotted horizontal and longitudinal axes indicate the lower thresholds for statistical (P < 0.05) and biological significance (2 fold change) of expression, respectively. **c** Specific fold changes for genes that were significantly regulated due to rehabilitation are presented. (One way ANOVA; significantly different than *3 days or †14 days post-VML)

To investigate cellular mechanisms of the VML injury pathophysiology, additional cohorts of injured mice were allocated to VML with no repair or early rehabilitation (ROM or ROM-E) for 3, 7, or 14 days following injury and transcriptional changes in inflammatory, neurogenic, fibrotic, myogenic, and metabolic genes were assessed (Additional file 1: Table S1). In the VML only group, there were 30 genes differentially regulated in the injured limb compared to the uninjured limb, independent of time (i.e., 3, 7, 14 days). Most all genes probed were significantly up-regulated over control tissue, notably only Mstn, Slc2a4, and Ppargc1a displayed down-regulation (Fig. 5 and Additional file 4: Table S2). Several of these genes demonstrated transient changes in differential regulation, as many myogenic, metabolic, and inflammatory genes were significantly up-regulated at 3 in comparison to both 7 and 14 days post-injury. A small number of fibrotic and neurogenic genes (Mmp9, Col3a1, Fbxo32, Tgfbr3, and Nrg1) were significantly up-regulated at 3 in comparison to only14 days. This supports a VML-related regulation of inflammatory genes which had at least a 4-fold increase at 3 days compared to both the 7 and 14 day time points, although notably inflammation was still significantly elevated at the later time points.

### Acute genetic response to rehabilitation

To determine how early rehabilitation approaches may alter the gene expression pattern associated with VML injury, gene expression of the gastrocnemius muscle (24 h following the final rehabilitation bout) from mice that underwent rehabilitation (ROM & ROM-E) were compared to non-repaired VML muscle at the same time points (3, 7, or 14 days post-VML). Both the ROM and ROM-E groups were collapsed into ‘early rehabilitation’ (Fig. 5). Significant differences in gene expression were observed at 3 days post-injury between VML only and early rehabilitation groups (ROM & ROM-E). There was a significant down-regulation in mostly inflammatory gene expression with rehabilitation at 3 days post-injury compared to other time points. In particular, inflammatory (Il33, Tnf, Il4, CxCr3, CxC1, Ccl5, Ccl2) and metabolic (Akt2, Prkaal, Pparfcla, Sk2a4) genes were down-regulated at 3 days post-injury. This suggests that early rehabilitation may mitigate the acute maladaptive response to VML injury, which may be related to the chronic improvements in muscle function and passive stiffness.

## Discussion

Current regenerative medicine and rehabilitation techniques for VML injured patients have not shown significant restoration of muscle strength or limb function [6]. A major limiting factor to rehabilitation is the remaining muscle tissue following injury, and its adaptability and/or capacity to recover from injury. Unfortunately, in VML-injured patients, rehabilitation often begins after significant fibrosis has occurred in the muscle unit, contributing to low functional improvements [37]. To overcome this timing limitation we investigated techniques (i.e., passive range of motion and electrical stimulation) that can begin early after VML injury. The most salient findings were that 1) early initiation of passive range of motion therapy attenuated injury-induced elevations of muscle stiffness, but did not improve active muscle function (Fig. 2), early co-delivery of neural electrical stimulation with passive range of motion therapy 2) improved active muscle function, but did not attenuate rising muscle stiffness (Fig. 2), and 3) abrogated the capacity of passive range of motion therapy to prevent injury induced elevations of muscle stiffness.

Skeletal muscle fibrosis is known to impede muscle healing and regeneration, alter the microenvironment of the muscle, and causes destruction of muscle architecture [38]. It is possible that the overwhelming fibrotic response after injury may be limiting rehabilitation and/or further impacting the remaining muscle as it is left to follow the natural sequela of injury. In pathologies such as Duchenne muscular dystrophy and cerebral palsy it has been proposed that the organization and structure of the fibrotic deposition may have a greater role in functional impairments than the total amount of collagen [39]. Because fibrotic tissue fills the void left by VML injury [4, 37], we initially hypothesized that early range of motion therapy, alone or in combination with electrical stimulation, would attenuated fibrotic tissue deposition and stiffness. The observation of reduced stiffness after range of motion therapy partially supports this hypothesis; however, we did not detect predictable differences among rehabilitation groups in terms of total collagen content in the injured limb (Fig. 4). This discrepancy is similar to prior observations of disconnect between collagen crosslinking characteristics with tissue stiffness [40] and supports further investigation of range of motion therapy impact on collagen type, organization, or structure of collagen. The notable positive impact of reduced passive muscle stiffness with range of motion therapy alone does stand to have translatable benefits for this patient population, in which even modest improvements to daily activities may have significant impact on patient quality of life.

Skeletal muscle metabolic capacity is highly plastic and under most conditions has the capability to regenerate after injury. Specifically, during the normal regenerative processes, such as occurring after traumatic myotoxic injury, mitochondrial biogenesis accompanies muscle recovery from injury and is likely necessary to meet the energy demands of muscle repair [41, 42]. Injuries such as VML present a non-recoverable injury, in which the muscle has limited regenerative potential and loses the ability to recover [7]. Mitochondrial function was less in VML-injured mice compared to completely uninjured (i.e., naïve) control mice, however there was no detectable differences in mitochondrial function between injured and contralateral uninjured muscles paired across the same animal. This finding raises several intriguing questions regarding the systemic and chronic effects of VML injury and possibility of low-grade systemic inflammation. Large-scale traumatic injuries such as burn traumas have been associated with low-grade, systemic inflammation that is reported to negatively influence mitochondrial function [43, 44]. Furthermore, following various neuromusculoskeletal injuries such as hip fracture there is noted systemic inflammation which is hypothesized to contribute to lack of muscle regeneration [45]. VML injury induction of acute and chronic systemic inflammation presents a potential pivotal component of the pathogenic response that when left untreated may worsen disability and may impede rehabilitative and regenerative treatment efficacy.

To date only a few studies have examined fibrotic and myogenic responses at 1 to 2 weeks following VML injury. Previous work has indicated that connective tissue growth mediated regulation through TGF-1β family gene expression is greater at 1 week following VML and at 2 weeks there is induction of myogenic and inflammatory genes [13, 46]. Unique to VML injury however is the duration of inflammatory gene induction, which appears to be both heightened and prolonged following injury [34, 47] compared to common endogenously healing injuries [48]. This work investigated the early genetic response to VML injury inflammatory, neurogenic, fibrotic, myogenic, and metabolic genes over the first 2 weeks post-VML. Few selected genes were down-regulated following VML injury alone; specifically Mstn, Slc2a4, and Ppargc1a downregulation occurred at all time points through 2 weeks post-injury. Primarily there was a substantial up-regulation of probed genes following VML injury. In particular inflammatory genes probed appear to most up-regulated at 3 days over 7 and 14 days post-VML. Notably the expression at both 7 and 14 days was still greatly up-regulated from uninjured muscle. Early rehabilitation appears to dampen this inflammatory response, especially at 3 days post-injury and future work should investigate how early rehabilitation may impact any systemic inflammation related to VML injury.

It stands to reason that VML-injured animals are expected to be less physically active compared to uninjured controls, which could produce a lower basal metabolic capacity. A current limitation for the field is an understanding of the physical or metabolic activity of patients with VML injury. Importantly though, VML-injured rodents are able to elevate physical activity as evidenced by increased voluntary wheel running distance [11–14], but the ability of the VML-injured limb or uninjured limb to positively adapt to the elevated physical activity in terms of metabolic capacity, balance in protein synthesis/degradation, and fiber type distributions is unknown and future work should begin to understand this complex relationship. Additionally, future work is needed to continue to understand the pathophysiologic state of the muscle remaining after VML injury with or without additional rehabilitation, as there is a significant need to understand potential therapeutic targets that could benefit the loss of function following VML injury. Collectively, limited and/or lost mobility, poor metabolic function, and/or low-grade systemic inflammation after VML injury may all contribute to development and/or exacerbation of metabolic syndrome and cardiovascular disease in patients with VML injury. Therefore, identifying therapeutic interventions that promote muscle health and physical activity may lessen the health burden and medical costs of VML injury.

## Conclusions

Many existing patients with a VML injury could benefit from more readily translatable strategies directed toward improving the remaining tissue, allowing them to engage in more daily actives, and improving quality of life. Strategies to improve the quality of the remaining muscle may also better prepare the individual to take advantage of advanced regenerative engineering approaches to regenerate tissue as they become available in the future. This work developed and evaluated early rehabilitation paradigms to understand the metabolic, genetic, and functional response of the remaining tissue after a multi-muscle VML injury, in efforts to improve the muscle remaining following injury. We expect that identifying genetic and functional plasticity in the remaining skeletal muscle with early rehabilitation approaches may facilitate evidenced-based practice at the clinical level following further translation. Herein we suggest that the remaining tissue following VML injury beneficially adapts to early rehabilitation, but that limitations in the metabolic plasticity of the muscle still exist.

## Additional files

Additional file 1: **Table S1.** Genes probed following VML injury and early rehabilitation. (DOCX 22 kb)

Additional file 2: **Figure S1.** Effect of VML injury and rehabilitation on study endpoint body mass and gastrocnemius muscle mass. **a** There was a main effect of time, independent group, for body mass indicating mice at 4 Month post-VML injury weighed significantly more (~ 6%) than mice at 1 Month and 2 Month post-VML injury. **b** There was a main effect of time, independent of group, for injured gastrocnemius muscle mass as a fraction of the contralateral uninjured control indicating mice at 4 Month post-VML injury had significantly more (~ 13%) proportional injured gastrocnemius muscle mass than mice at 1 Month. (JPG 839 kb)

Additional file 3: **Figure S2.** Representative torque-time waveforms during peak isometric contraction from 4 Month VML, ROM, and ROM-E groups compared to completely uninjured controls. The rate of relaxation for all terminal peak isometric contractions was evaluated. The rate of relaxation was greater following ROM-E rehabilitation compared to VML-alone and ROM rehabilitation, independent of time. Control = 576 ± 34 mN●m sec − 1. (JPG 99 kb)

Additional file 4: **Table S2.** Fold change in gene expression (vs. control) following VML injury. (DOCX 30 kb)

## Abbreviations

E: Intermittent electrical stimulation
Pt-Ir: Platinum-iridium
ROM: Range of motion
VML: Volumetric muscle loss
