# Supplementary material for: Early rehabilitation for volumetric muscle loss injury augments endogenous regenerative aspects of muscle strength and oxidative capacity
Source: BMC Musculoskelet Disord. 2018 May 29;19:173. doi: 10.1186/s12891-018-2095-6 (PMC5975473; doi:10.1186/s12891-018-2095-6)
Supplement: Supplementary file 1 — Table S1. Genes probed following VML injury and early rehabilitation. (DOCX 22 kb) [file 12891_2018_2095_MOESM1_ESM.docx]

| **Table S1.** Genes probed following VML injury and early rehabilitation | | |
| --- | --- | --- |
| ***Classification*** | **Gene** | **Name** |
| ***Inflammatory*** | Cxcl10 | chemokine (C-X-C motif) ligand 10 |
|  | Cxcr3 | chemokine (C-X-C motif) receptor 3 |
|  | Ccl2 | chemokine (C-C motif) ligand 2 |
|  | Ccl5 | chemokine (C-C motif) ligand 5 |
|  | Cxcl1 | chemokine (C-X-C motif) ligand 1 |
|  | Ifng | interferon gamma |
|  | Il1a | interleukin 1 alpha |
|  | Il1b | interleukin 1 beta |
|  | Il4 | interleukin 4 |
|  | Il6 | interleukin 6 |
|  | Il10 | interleukin 10 |
|  | Il33 | interleukin 33 |
|  | Tgfb1 | transforming growth factor, beta 1 |
|  | Tnf | tumor necrosis factor |
| ***Myogenic*** | Mstn | myostatin |
|  | Myod1 | myogenic differentiation 1 |
|  | Myog | myogenin |
|  | Pax7 | paired box 7 |
| ***Neurogenic*** | Bdnf | brain derived neurotrophic factor |
|  | Nrg1 | neuregulin 1 |
| ***Metabolic*** | Akt1 | thymoma viral proto-ongogene 1 |
|  | Akt2 | thymoma viral proto-oncogene 2 |
|  | Capn2 | calpain 2 |
|  | Casp3 | caspase 3 |
|  | Cox4i1 | cytochrome c oxidase subunit IV isoform 1 |
|  | Foxo1 | forkhead box O1 |
|  | Foxo3 | forkhead box O3 |
|  | Fbxo32 | F-box protein 32 |
|  | Ndufa11 | NADH dehydrogenase (ubiquinone) 1 alpha subcomplex 11 |
|  | Pparg | peroxisome proligerator activated receptor gamma |
|  | Ppargc1a | peroxisome proliferative activated receptor, gamma, coactivator 1 alpha |
|  | Ppargc1b | peroxisome proliferative activated receptor, gamma, coactivator 1 beta |
|  | Prkaa1 | protein kinase, AMP-activated, alpha 1 catalytic subunit |
|  | Rps6kb1 | ribosomal protein S6 kinase, polypeptide 1 |
|  | Sdhb | succinate dehydrogenase complex, subunit B, iron sulfur (Ip) |
|  | Slc2a4 | solute carrier family 2 (facilitated glucose transporter), member 4 |
|  | Tfam | transcription factor A, mitochondrial |
|  | Trim63 | tripartie motif-containing 63 |
| ***Fibrotic*** | Col1a1 | collagen, type I, alpha 1 |
|  | Col3a1 | collagen, type III, alpha 1 |
|  | Mmp9 | matrix metallopeptidase 9 |
|  | Tgfbr3 | transforming growth factor, beta receptor III |

Genes used in the custom designed mouse specific PCR array
