# Supplementary figures and images for: Early rehabilitation for volumetric muscle loss injury augments endogenous regenerative aspects of muscle strength and oxidative capacity
Source: BMC Musculoskelet Disord. 2018 May 29;19:173. doi: 10.1186/s12891-018-2095-6 (PMC5975473; doi:10.1186/s12891-018-2095-6)

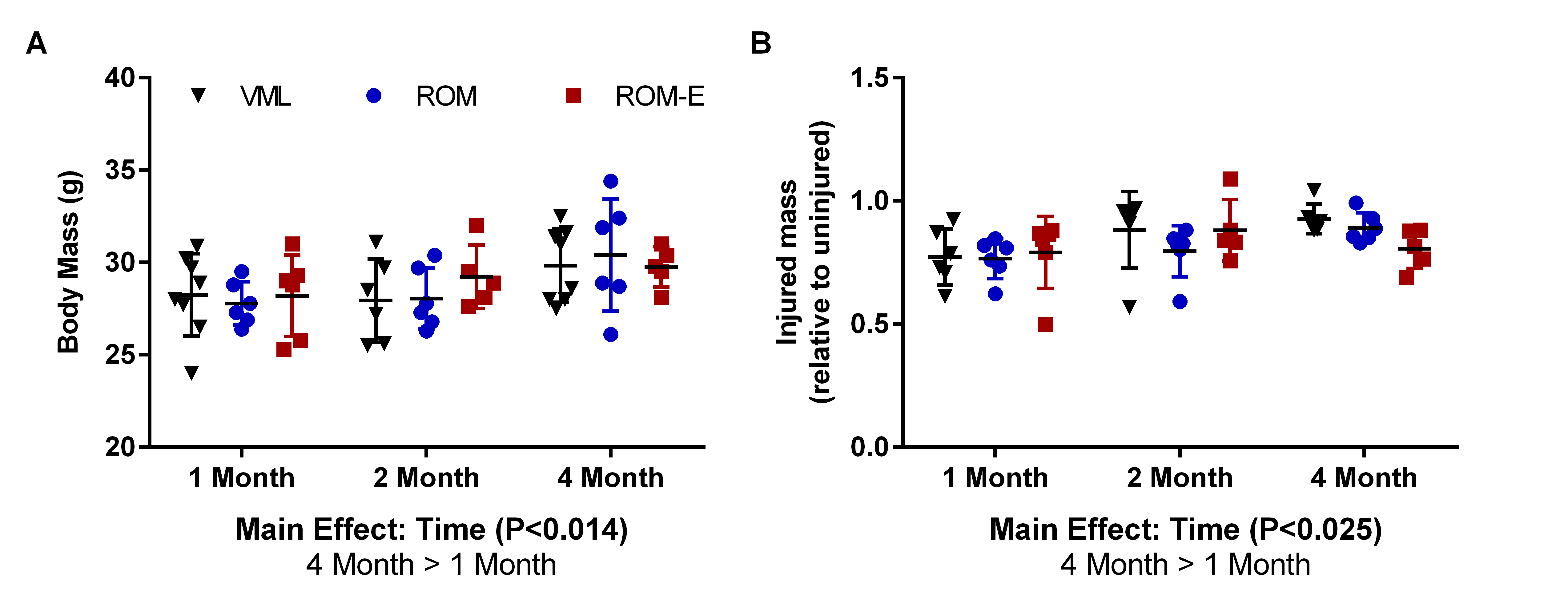

Supplement: Supplementary file 2 — Figure S1. Effect of VML injury and rehabilitation on study endpoint body mass and gastrocnemius muscle mass. a There was a main effect of time, independent group, for body mass indicating mice at 4 Month post-VML injury weighed significantly more (~ 6%) than mice at 1 Month and 2 Month post-VML injury. b There was a main effect of time, independent of group, for injured gastrocnemius muscle mass as a fraction of the contralateral uninjured control indicating mice at 4 Month post-VML injury had significantly more (~ 13%) proportional injured gastrocnemius muscle mass than mice at 1 Month. (JPG 839 kb) [file 12891_2018_2095_MOESM2_ESM.jpg]

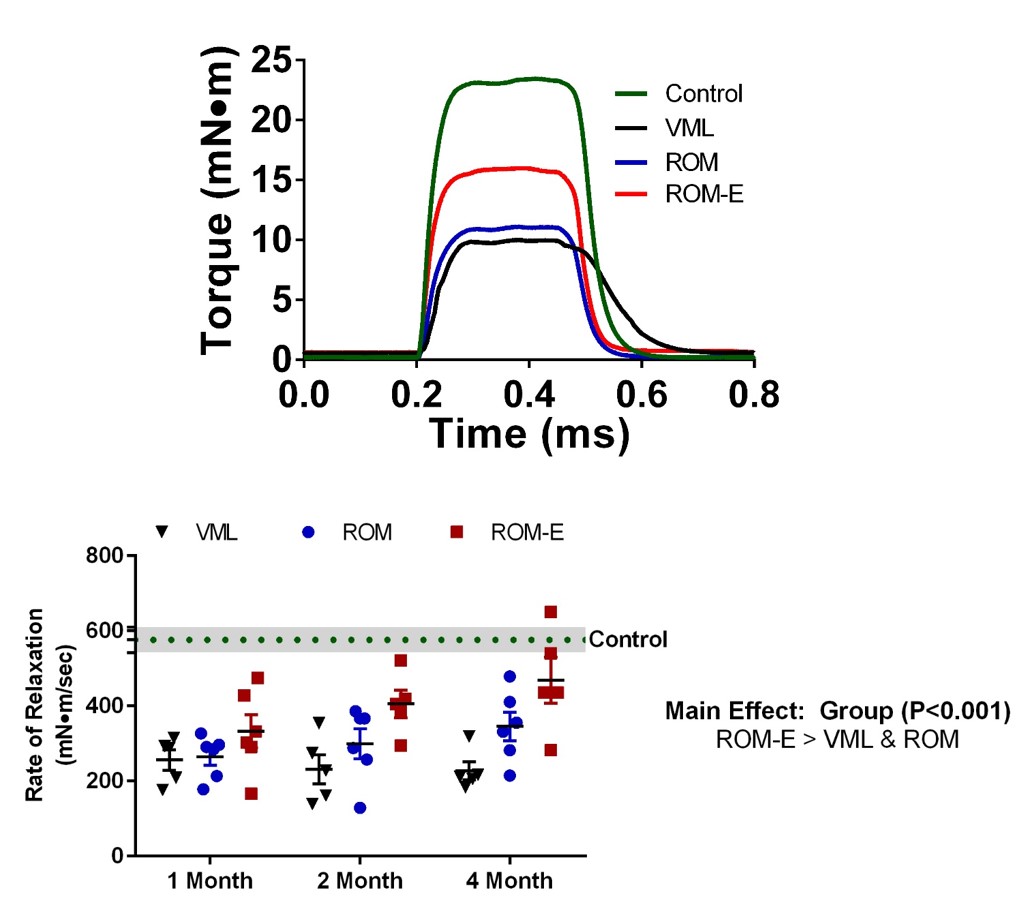

Supplement: Supplementary file 3 — Figure S2. Representative torque-time waveforms during peak isometric contraction from 4 Month VML, ROM, and ROM-E groups compared to completely uninjured controls. The rate of relaxation for all terminal peak isometric contractions was evaluated. The rate of relaxation was greater following ROM-E rehabilitation compared to VML-alone and ROM rehabilitation, independent of time. Control = 576 ± 34 mN●m sec − 1. (JPG 99 kb) [file 12891_2018_2095_MOESM3_ESM.jpg]
