# Supplementary material for: Early rehabilitation for volumetric muscle loss injury augments endogenous regenerative aspects of muscle strength and oxidative capacity
Source: BMC Musculoskelet Disord. 2018 May 29;19:173. doi: 10.1186/s12891-018-2095-6 (PMC5975473; doi:10.1186/s12891-018-2095-6)
Supplement: Supplementary file 4 — Table S2. Fold change in gene expression (vs. control) following VML injury. (DOCX 30 kb) [file 12891_2018_2095_MOESM4_ESM.docx]

| **Table S2.** Fold change in gene expression (vs. control) following VML injury | | | | | | | | | | | |
| --- | --- | --- | --- | --- | --- | --- | --- | --- | --- | --- | --- |
| **Gene** | **3 days** |  |  | **7 days** |  |  | **14 days** |  |  | **One way ANOVA p-value** | |
| ***Inflammatory*** | |  |  |  |  |  |  |  |  |  |  |
| Cxcl10 | 62.4 | ± | 15.2 | 17.8 | ± | 1.9 | 12.7 | ± | 4.0 | **0.007** | *3 > 7 & 14 days* |
| Cxcr3 | 43.8 | ± | 7.4 | 20.1 | ± | 3.0 | 16.1 | ± | 6.7 | **0.021** | *3 > 7 & 14 days* |
| Ccl2 | 336.9 | ± | 65.3 | 13.2 | ± | 3.7 | 3.9 | ± | 1.1 | **0.001** | *3 > 7 & 14 days* |
| Ccl5 | 32.4 | ± | 14.1 | 30.8 | ± | 4.5 | 34.7 | ± | 11.9 | 0.969 |  |
| Cxcl1 | 144.9 | ± | 16.5 | 10.9 | ± | 6.0 | 2.3 | ± | 0.8 | **<0.001** | *3 > 7 & 14 days* |
| Ifng | 10.3 | ± | 2.4 | 6.2 | ± | 2.5 | 15.1 | ± | 6.3 | 0.375 |  |
| Il1a | 12.3 | ± | 2.7 | 4.7 | ± | 0.4 | 2.0 | ± | 0.3 | **0.004** | *3 > 7 & 14 days* |
| Il1b | 252.2 | ± | 35.0 | 46.5 | ± | 28.4 | 5.1 | ± | 1.6 | **0.001** | *3 > 7 & 14 days* |
| Il4 | 5.5 | ± | 1.3 | 4.3 | ± | 0.6 | 2.0 | ± | 1.0 | 0.090 |  |
| Il6 | 104.8 | ± | 9.5 | 17.0 | ± | 12.3 | 1.9 | ± | 0.7 | **<0.001** | *3 > 7 & 14 days* |
| Il10 | 167.5 | ± | 45.5 | 18.5 | ± | 3.5 | 14.7 | ± | 5.4 | **0.004** | *3 > 7 & 14 days* |
| Il33 | 14.8 | ± | 1.6 | 3.5 | ± | 0.5 | 2.3 | ± | 0.4 | **<0.001** | *3 > 7 & 14 days* |
| Tgfb1 | 38.7 | ± | 4.4 | 10.4 | ± | 1.6 | 7.9 | ± | 2.4 | **<0.001** | *3 > 7 & 14 days* |
| Tnf | 95.2 | ± | 23.9 | 11.2 | ± | 2.7 | 6.9 | ± | 2.7 | **0.002** | *3 > 7 & 14 days* |
| ***Myogenic*** | |  |  |  |  |  |  |  |  |  |  |
| Mstn | 0.5 | ± | 0.1 | 0.4 | ± | 0.0 | 0.4 | ± | 0.0 | 0.077 |  |
| Myod1 | 14.4 | ± | 0.8 | 3.9 | ± | 0.7 | 2.8 | ± | 0.3 | **<0.001** | *3 > 7 & 14 days* |
| Myog | 62.5 | ± | 2.6 | 19.5 | ± | 4.6 | 9.6 | ± | 2.5 | **<0.001** | *3 > 7 & 14 days* |
| Pax7 | 11.0 | ± | 1.5 | 4.5 | ± | 1.0 | 2.8 | ± | 0.6 | **0.001** | *3 > 7 & 14 days* |
| ***Neurogenic*** |  |  |  |  |  |  |  |  |  |  |  |
| Bdnf | 1.6 | ± | 0.5 | 3.1 | ± | 0.4 | 2.2 | ± | 0.5 | 0.148 |  |
| Nrg1 | 139.3 | ± | 16.1 | 80.2 | ± | 24.2 | 12.6 | ± | 6.4 | **0.002** | *3 > 14 days* |
| ***Metabolic*** |  |  |  |  |  |  |  |  |  |  |  |
| Akt1 | 9.8 | ± | 0.8 | 3.7 | ± | 0.7 | 2.2 | ± | 0.5 | **<0.001** | *3 > 7 & 14 days* |
| Akt2 | 2.3 | ± | 0.1 | 1.5 | ± | 0.2 | 1.6 | ± | 0.5 | 0.235 |  |
| Capn2 | 7.5 | ± | 0.5 | 2.5 | ± | 0.3 | 1.9 | ± | 0.3 | **<0.001** | *3 > 7 & 14 days* |
| Casp3 | 31.9 | ± | 4.9 | 11.3 | ± | 3.0 | 5.2 | ± | 1.0 | **0.001** | *3 > 7 & 14 days* |
| Cox4i1 | 1.7 | ± | 0.2 | 1.9 | ± | 0.4 | 1.6 | ± | 0.5 | 0.882 |  |
| Foxo1 | 3.1 | ± | 0.4 | 2.5 | ± | 0.3 | 2.3 | ± | 0.6 | 0.430 |  |
| Foxo3 | 3.8 | ± | 0.7 | 2.0 | ± | 0.4 | 1.9 | ± | 0.5 | 0.058 |  |
| Fbxo32 | 1.6 | ± | 0.1 | 1.0 | ± | 0.2 | 0.9 | ± | 0.2 | **0.031** | *3 > 14 days* |
| Ndufa11 | 1.3 | ± | 0.1 | 1.5 | ± | 0.2 | 1.5 | ± | 0.4 | 0.892 |  |
| Pparg | 8.5 | ± | 2.0 | 3.3 | ± | 0.4 | 2.9 | ± | 0.9 | **0.022** | *3 > 7 & 14 days* |
| Ppargc1a | 0.6 | ± | 0.1 | 0.6 | ± | 0.1 | 0.7 | ± | 0.0 | 0.330 |  |
| Ppargc1b | 1.9 | ± | 0.1 | 1.7 | ± | 0.1 | 1.8 | ± | 0.5 | 0.929 |  |
| Prkaa1 | 6.5 | ± | 0.7 | 2.7 | ± | 0.3 | 2.1 | ± | 0.5 | **0.001** | *3 > 7 & 14 days* |
| Rps6kb1 | 3.9 | ± | 0.7 | 2.0 | ± | 0.3 | 1.6 | ± | 0.4 | **0.019** | *3 > 7 & 14 days* |
| Sdhb | 0.9 | ± | 0.1 | 1.2 | ± | 0.3 | 1.2 | ± | 0.3 | 0.589 |  |
| Slc2a4 | 0.5 | ± | 0.1 | 0.4 | ± | 0.0 | 0.6 | ± | 0.0 | 0.088 |  |
| Tfam | 2.1 | ± | 0.3 | 1.6 | ± | 0.2 | 1.6 | ± | 0.5 | 0.414 |  |
| Trim63 | 3.5 | ± | 0.1 | 1.6 | ± | 0.3 | 1.1 | ± | 0.2 | **<0.001** | *3 > 7 & 14 days* |
| ***Fibrotic*** |  |  |  |  |  |  |  |  |  |  |  |
| Col1a1 | 17.1 | ± | 1.7 | 27.3 | ± | 3.8 | 16.7 | ± | 5.0 | 0.129 |  |
| Col3a1 | 23.9 | ± | 2.9 | 27.7 | ± | 2.8 | 15.2 | ± | 2.7 | **0.032** | *3 > 14 days* |
| Mmp9 | 61.9 | ± | 8.7 | 23.0 | ± | 8.0 | 2.6 | ± | 1.5 | **<0.001** | *3 > 14 days* |
| Tgfbr3 | 4.7 | ± | 0.8 | 2.7 | ± | 0.5 | 2.2 | ± | 0.2 | **0.023** | *3 > 14 days* |

Custom designed mouse specific PCR array investigated inflammatory, myogenic, neurogenic, metabolic, and fibrotic transcriptional response acutely after VML injury, gene expression fold changes are relative to contralateral control muscle. Bold font indicates significance and ANOVA differences are noted; fold change values are means±SE
